# Book Reviews

**Published:** 1915-11

**Authors:** 


### BOOK REVIEWS

**Books mentioned may be inspected at and ordered through this office. So far as possible, books received in any month will be reviewed in the issue of the second month following.**

---

**Histology.** Rudolph Krause, Berlin. Translated from an original Mss. and printed only in English. Published by the Rebman Co., N. Y., 274 pages, 36 illustrations (mostly diagrammatic) with numbered references to colored illustrations in the author's "Course in Normal Histology." $2.50.

This book discusses histology uunder the general classification of the cell, the tissues and the microscopic anatomy of organs. It is concisely written and adapted to quick reference by headings, black letters and italics. While obviously, the student is dependent upon the other book mentioned, or must refer to some other source of illustration, to actual slides or must draw upon his memory, the separate publication of this work is economic and, for reference and review, time and labor saving.

---

**American Urological Assn.** Transactions of 13th Annual Meeting at Philadelphia, June 18-20, 1914. Publication Committee: Hugh Cabot, Richard Frothingham O'Neil, George Gilbert Smith. Printed at the Riverdale Press, Brookline, Mass. 327 pages with plates.

A valuable collection of monographs. In accordance with our custom, being unable to mention all papers in such transactions, we make no attempt at a review.

---

**Potter's Compend of Human Anatomy.** 8th edition, revised by D. Gregg Metheny, M. D., L. C. P., & S., L. F. P. S., of Philadelphia. Published by P. Blakiston's Son & Co., Philadelphia. 428 pages, 139 illustrations, tables and 16 plates of arteries and nerves. $1.00.

This is one of the familiar brown Quiz Compends, which we remember as a help in student days. Beteween the covers, the aspect is not so familiar. The rebuke of a teacher to a student insistent on an up-to-date text book: Very few new bones have been discovered in the last twenty years'' may be correct, but very many improvements have been devised in the methods of impressing them on the memory. We are reminded of the flight of time by the transference of S. O. L. Potter's name from the place of author to the title.

---

**Progressive Medicine,** edited by H. A. Hare and L. F. Appleman, Philadelphia, quarterly, $6.00 per year. Published by Lee & Febiger, Philadelphia and N. Y.

Vol. 18, No. 3, Sept. 1, 1915, deals with the thorax and viscera, including the thyroid (Wm. Ewart), Dermatology and Syphilis (Wm. S. Gottheil), Obstetrics (Edward P. Davis, Diseases of the Nervous System (Wm. G. Spiller). The influence of the European War is noted in several instances. This series shows steady improvement, notably in the brevity and variety of reviews and in the more liberal notice of articles in current medical literature.

---

**The Clinics of John B. Murphy, M. D.** Published bi-monthly by the W. B. Saunders Co. of Philadelphia, $8.00 per year.

Vol. 4, No. 4, Aug. 1915, includes pages 579-795 of the current volume and deals with a great variety of surgical topics.

**The Medical Clinics of Chicago.** Published bi-monthly by the W. B. Saunders Co., of Philadelphia. $8.00 per year.

Vol. 1, No. 2, Sept. 1915, includes pages 209-403, represents several authors and various topics in medicine.

---

**Marie Tornowska.** Mrs. Anne Vivanti Chartres, Published by the Century Co., N. Y., 303 pages, $1.50.

This is a biography, one might almost say an edited autobiography, of a Russian countess. Her husband fatally wounds her first lover; with whom she spends a hundred days of necessarily platonic affection while he vainly seeks medical and surgical skill to avert death. Her second lover conspires with her to use her fourth lover as a tool to secure the death of the third, who carries life insurance in her favor. She is sentenced to eight years in prison. The book is a novel with a purpose—to secure her social pardon and leniency in similar cases. From this bald statement of the plot, such a purpose may not appear reasonable but, here we strike the interest of the book to the medical profession. The heroine is obviously hysteric, with a double neuropathic heredity. Epilepsy is implied but scarcely established in her own case, though one need not question the history of epilepsy on the part of the mother. In addition, gynaecologic and metabolic disturbances are somewhat vaguely claimed, and the use of morphine, first administered by a physician himself the victim of this habit, is definitely stated. Considered merely as a story, the book is thrilling though hardly of a desirable type, and it well illustrates the frequent surpassing of verisimilitude by fact. One may not agree with the purpose of the book, which is endorsed by L. M. Bozzi of Genoa, a medical expert at her trial, but the medical and penologic problems are worth considering.

---

**Physical Diagnosis.** Dr. Richard C. Cabot, Boston. Published by Wm. Wood & Co., N. Y., 6th edition, 521 pages, 6 plates and 268 figures. $3.25.

This work bears little resemblance to the older ones that dealt with inspection, palpation, auscultation, percussion, succussion, and that devoted a few pages to everything except the heart and lungs. All parts of the body are considered and the methods of physical exploration are introduced in their natural places just as the examination of the blood, electric and other tests of reflexes are given a place. With the addition of the X-ray to our diagnostic armamentarium, and the broadening of the scope of physical diagnosis, inspection assumes a far greater importance and a higher standard of scientific accuracy. We venture to disagree with the statement that auscultatory percussion should not proceed in a radial direction from the stethoscope. It is only by following this plan that an area can be quickly and sharply mapped, and while it is true that the sound increases in volume as the stethoscope rests to another unit of conduction is, under normal conditions, so abrupt as to be unmistakable. The substitution of a tuning fork or electric buzzer for the percussing finger, often gives better results. As in his other books, Cabot has introduced an element of originality even in discussing familiar problems. He is a genius in relating and correlating facts, and thus giving a wider significance and practical value to any one known principle or observation.

The best review of this book, however, is what the intelligent reader will say to himself over and over again: ''Why, that's so, but I never thought of it in that way before.''

---

**Students' Text Book of Hygiene.** W. James Wilson, M. D., D. Sc., D. P. H., Belfast. Published by the Rebman Co., N. Y. 270 pages $2.50.

This book is based on the author's lecture course at Queen's University and, in many details, is limited to British experience and needs. The chapter on Infection and Immunity, though brief, is lucid. In discussing heredity and eugenics, it is stated that the strictly hereditary diseases are forms of nervous disease-haemophilia, Daltonism, albinism, myopia and ichthyosis, and that the trait appears only in males but is transmitted through females who appear normal, the phenomenon being due to the absence of a special determiner from the male sex chromosome.

In speaking of the disposal of refuse, it is stated that cremation is the ideal method but expensive—25 cents a ton—a destructor unit capable of burning 10-15 tons a day being sufficient for a population of 10,000. By implication, superficial burial is favored, on account of the greater number of sarcophagic bacteria in the top layer of soil, one foot being sufficient to prevent a nuisance. However, the English sanitary laws require depths of 3 feet to the top of the coffin for children under 12, and 4 feet for older persons. One acre is stated to be a fair estimate of burial space needed for a community of 1,000 for 14 years. This is a surprisingly liberal estimate. The annual death rate for England is between 14 and 15: 1,000, say 200 burials in 14 years. This allows 267 square feet to a burial.

The chapter on vital statistics includes some mathematic considerations often overlooked and extremely valuable as preventing fallacious conclusions.

**The Book of the Fly.** G. Hurlstone Hardy. Published by the Rebman Co., N. Y. 124 pages, illustrated. $0.80.

While presented in rather non-technical style, the matter is really technical, especially in the appendix, about a quarter of the whole volume which deals with zoologic classification, anatomy, etc. The biology, relation to filth and disease, methods of exclusion and extermination are treated quite in detail.

---

**Twelve Lectures on the Treatment of Gonorrhoea in the Male.** Dr. P. Asch of Strassburg, translated and annotated by Dr. Faxton E. Gardner of N. Y. Published by the Rebman Co., N. Y. 104 pages, illustrated, $1.00.

The author is sceptic as to the communication of gonorrhoea by inanimate objects and thinks that variations in the incubation period, from 12 hours to 2 or 3 weeks due to differences in virulence explain most of the apparent exceptional means of conveyance. Buccal diplococci, degeneration into single cocci and even into Gram-positive forms are recognized. Complications are duly considered and endoscopic methods of diagnosis and treatment are discussed quite in detail.

---

**The Starvation Treatment of Diabetes** with a Series of Graduated Diets as used at the Massachusetts General Hospital, by Lewis Webb Hill, M. D. and Rena S. Eckman, Dietitian, with an introduction by Richard C. Cabot, M. D. Price $1.00. W. M. Leonard, Publisher.

This book furnishes to the general practitioner in compact form the details of the latest and most successful treatment of Diabetes Mellitus. It presents the clinical application of the work done in recent years by Dr. Allen at Harvard and the Rockefeller Institute.

This method of treatment, carried out by Dr. Allen at the Rockefeller Hospital, and the staff at the Massachusetts General Hospital, has proved very successful. As Dr. Cabot states in the introduction: "It seems already clearly proven that Dr. Allen has notably advanced our ability to combat the disease. * * * To all who wish to give their patients the benefit of this treatment I can heartily recommend this book." In a recent address Dr. Allen said, "However specialists may feel there is no doubt that a majority of average practitioners feel bewildered and helpless concerning Diabetes."

To all who have been tried by this baffling disease, this little volume, with its description of treatment, tests and diets will be of greatest service.

